# Habituation, Adaptation and Prediction Processes in Neurodevelopmental Disorders: A Comprehensive Review

**DOI:** 10.3390/brainsci13071110

**Published:** 2023-07-21

**Authors:** Annabelle Merchie, Marie Gomot

**Affiliations:** UMR 1253 iBrain, Université de Tours, INSERM, 37000 Tours, France; annabelle.merchie@inserm.fr

**Keywords:** habituation, neural adaptation, development, neurodevelopmental disorders

## Abstract

Habituation, the simplest form of learning preserved across species and evolution, is characterized by a response decrease as a stimulus is repeated. This adaptive function has been shown to be altered in some psychiatric and neurodevelopmental disorders such as autism spectrum disorder (ASD), attention-deficit/hyperactivity disorder (ADHD) or schizophrenia. At the brain level, habituation is characterized by a decrease in neural activity as a stimulation is repeated, referred to as neural adaptation. This phenomenon influences the ability to make predictions and to detect change, two processes altered in some neurodevelopmental and psychiatric disorders. In this comprehensive review, the objectives are to characterize habituation, neural adaptation, and prediction throughout typical development and in neurodevelopmental disorders; and to evaluate their implication in symptomatology, specifically in sensitivity to change or need for sameness. A summary of the different approaches to investigate adaptation will be proposed, in which we report the contribution of animal studies as well as electrophysiological studies in humans to understanding of underlying neuronal mechanisms.

## 1. Introduction

Habituation is an essential behavioral process found in all individuals. It allows individuals to adapt to their environment, by being less focused on an irrelevant repeated stimulus, and to detect and respond more quickly to change. This process requires the construction and constant updating of a sensory memory trace following the presentation of a stimulus, through which a repeated stimulus will be considered as regular [1]. A possible processing link to habituation has been described at the neural level, neural adaptation, which results in a decrease in neuronal activity upon repeated presentation of the same stimulus, usually referred to as repetition suppression (RS). Studies on RS were first conducted on animals, revealing this phenomenon at the level of the individual neuron, and then in humans, through function magnetic resonance imagery (fMRI) [2,3], and electroencephalography (EEG) [4,5]. Another phenomenon complementary to RS has been described at the neural level, repetition enhancement (RE), defined as an increase in the neural response with an increase in the number of repetitions, reflecting the anticipation and the expectation of the stimulus appearance [6]. RS and RE, together with prediction error (i.e., difference between the expectancy and the sensory stimuli [7]), are involved in prediction processes. Individuals with neurodevelopmental or psychiatric disorders such as autism spectrum disorder (ASD) or schizophrenia have previously been observed to exhibit atypical behavioral habituation [8,9], and, several studies have also shown atypical adaptation at the neural level [10] and possible atypical prediction processes [11].

The purpose of this comprehensive review is to describe the processes of behavioral habituation, neural adaptation, and prediction and how they have been studied throughout typical development. Adaptation and habituation have been studied extensively, but we will present and discuss these processes in this review more specifically in light of the more recent theory of predictive coding. Then, we will discuss their maturation and how they are impacted in three neurodevelopmental and psychiatric disorders: autism spectrum disorder (ASD), schizophrenia, and attention-deficit/hyperactivity disorder (ADHD).

## 2. From Behavioral Habituation to Neural Adaptation and Prediction Processes

### 2.1. Behavioral Habituation

Habituation is a behavioral process that has been studied for at least 50 years [12,13] and considered as a simple form of learning [13,14,15]. It is defined as a decrease in the behavioral response to a repeated stimulus [15]. In animals, habituation has been widely studied in *C. elegans* [16], in rat [17], in *Aplysia* [18] and in many other species (see Rankin et al., 2009 for a review [15]). In humans, habituation has been described in several modalities such as auditory [19,20] and smell [21]. Habituation has been extensively studied in children, but not as many studies have specifically investigated it in adults. A description of the processes during typical development will be given in Section 3.1.

The study of the neural mechanism underlying behavioral habituation are essential to describe the link between habituation and neural adaptation.

### 2.2. Repetition Suppression (RS)

RS is a cerebral phenomenon reflecting neural adaptation upon presentation of a repeated stimulus. It is defined as a decrease in neural response as the number of repetitions of the same stimulus increases [6,22,23]. Three explanatory models of RS have been proposed in the literature (see Grill-Spector et al. 2006, for an overview [23]).

**Figure 1:**
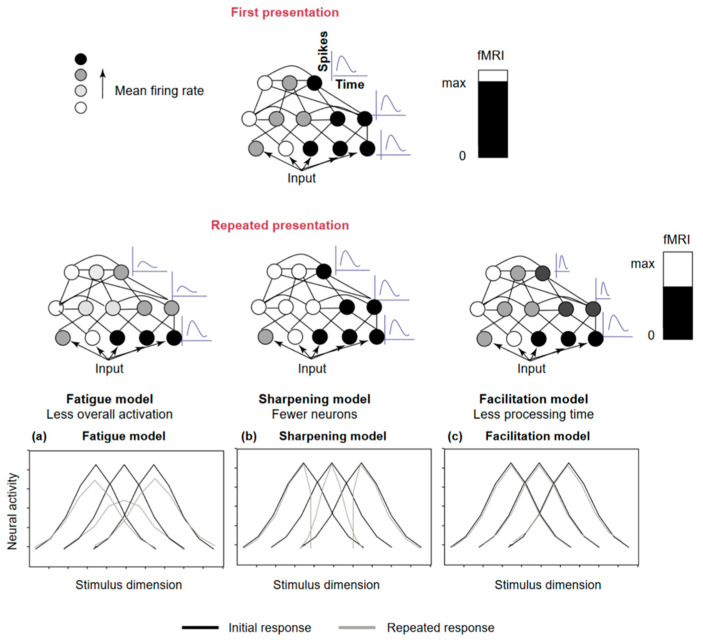
Representation of the three models that explain repetition suppression according to the review of Grill-Spector et al. (**a**) the Fatigue model: with repetition, the overall response will decrease in proportion to the initial response, but there will be no change in the preferred stimulus and tuning width., (**b**) the Sharpening model: Repeating the stimulus will lead to a reduction in the tuning bandwidth, resulting in a more focused response centered on the preferred stimulus, (**c**) The Facilitation model: does not offer specific or definitive predictions for changes in tuning curves. (Reprinted from “Repetition and the brain: Neural models of stimulus-specific effects”. Trends in Cognitive Sciences, 10(1), 14–23, Grill-Spector, K., Henson, R., and Martin, A. (2006), with permission from Elsevier) [23].

(1) The fatigue model, according to which each neuron that initially responds to the repeated stimulus sees its response decrease in proportion to its initial involvement. Thus, the stronger the initial response of a neuron, the more its response decreases with repetition. This results in a general decrease in response without modification of its latency or organization (Figure 1a) [24,25].

(2) The sharpening model, in which only the neurons responding to the stimulus and coding for irrelevant features see their response decrease with the repeated presentation of the same stimulus. Therefore, neurons that do not decrease their response at all, or soon after the presentation of a stimulus, will be more responsive to that stimulus (Figure 1b) [22,26].

(3) The facilitation (or accumulation) model where the repetition of a stimulus would lead to faster processing of that stimulus by the neurons involved, resulting in a shorter latency or response time (Figure 1c) [27].

RS has been studied in both animals, using single-cell recording, and humans, using fMRI, magnetoencephalography (MEG) and EEG.

#### 2.2.1. In Animals

Initially, studies revealed RS in animals at the individual neuron level. This phenomenon, called stimulus-specific adaptation (SSA), corresponds to a decrease in the response of a single neuron with an increase in the number of repetitions of the same stimulus (standard) without a decrease in the response to rare stimuli (deviant) [28]. RS was first described in the inferior temporal cortex (IT) of macaque [29,30,31,32,33,34,35,36,37,38,39] and was also found in the rat primary visual cortex [40]. Another study conducted in the IT of macaques also revealed a modulation of the RS by GABA_A_-mediated inhibition [41].

In the auditory modality, the first study observing SSA was conducted in the primary auditory cortex (AC1) and the medial geniculate body (MGB) of cats [42]. A decrease in the response of some neurons during repetition and a larger response to deviant non-impacted by repetition were found only in the AC1. Subsequently, other studies have shown SSA in the AC in other species [43,44,45,46,47]. At the subcortical level, several auditory studies highlighted SSA both in the inferior colliculus (IC) [48,49,50,51] and in the medial geniculate body (MGB) [52,53,54,55]. Some of these studies were interested in the involvement of AC in the generation of subcortical SSA and reported remaining SSA even after AC deactivation, although some of the subcortical neurons displayed reduced SSA [48,54], reflecting the involvement of AC in the modulation of subcortical SSA. Some of the studies in rodents highlighted the involvement of GABA_A_-mediated inhibition on the SSA, showing especially a reduced SSA after application of an antagonist of GABA_A_ receptors [51,55] or an increased SSA after application on an agonist of GABA_A_ receptors [55] suggesting the modulation of SSA by GABA_A_-mediated inhibition.

The SSA is thus a reliable neural phenomenon across species that has been observed, at both the cortical and the subcortical lever, in both auditory and visual modalities.

#### 2.2.2. In Humans

**Figure 2:**
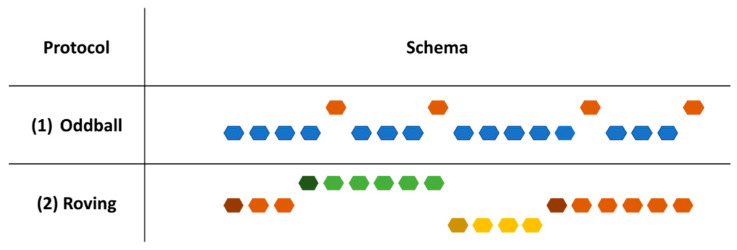
Representation of the different types of paradigms evoked in this review to study neural adaptation (repetition suppression). (1) Classic oddball sequence, in blue: standard, in orange: deviant. (2) Roving sequence: a stimulus is repeated several times followed by another stimulus; the first stimulus of each train (darker in the schema) corresponds to the deviant stimulus.

In humans, many studies have first examined the effects of repetition indirectly through the study of change detection [56,57]. For that, studies were conducted using oddball paradigms (see Figure 2) in which a repeated stimulus, called standard, is occasionally replaced by a new and different stimulus, called deviant. Results from these studies have focused on the response to the deviant stimulus, which reflect violation detection. In fMRI studies, change detection is reflected by an increase in brain activity in response to the deviant compared to the standard stimulus [58,59]. In EEG studies it is reflected by mismatch negativity (MMN), a negative component between 150 and 200 ms obtained by subtracting the response following the repetition of a standard stimulus to the response evoked by a changing deviant stimulus [60]. MMN thus appears to occur when a stimulus is incongruent with the memory representation of the preceding repeated stimuli [61,62], making it an indirect index of neuronal adaptation. However, recent studies have focused directly on the process reflecting the encoding of the repeated stimulus, the RS [57,58,63].

Although oddball paradigms are still used in some EEG studies [57], another type of paradigm more suitable for studying RS has been created, the roving paradigm, in which a stimulus is repeated *n* times and then followed by a new stimulus, which is also repeated *n* times (Figure 2). This type of paradigm leads to a continuous update of the memory trace that is suppressed at the end of each stimulus train [4,5,64,65,66]. Using this type of paradigm in the auditory modality, electrophysiological studies have highlighted that neuronal adaptation, in response to repeated sounds, appears to result in a combined decrease in the negative N1 component (a negative deflection of the response around 100 ms) and an increase in the positive P1 and P2 components (positive deflections of the response, respectively, around 100 and 200 ms), reflecting the adaptation of the response following the repeated presentation of a stimulus. By comparing the responses to a small number of repetitions of a new stimulus to the same stimulus after several repetitions, it was possible to isolate an electrophysiological index of auditory regularity encoding, repetition positivity (RP), which corresponds to a positive deflection between 50 and 250 ms, which increases as the number of repetitions increases [4,5,66,67]. Roving paradigms have also shown an “MMN memory trace effect”, i.e., an increase in MMN amplitude with an increase in the number of repetitions [4,5,66,68,69], reinforcing the idea that MMN would be an indirect index of adaptation. Moreover, it has been suggested that RP could be linked to SSA observed in animals [5,42,67].

RP has been observed for sounds of different natures: pure tones [4,56,67,68,70], or more complex acoustic sounds such as vocal sounds [71,72]. However, the complexity and the quantity of information to encode appears to influence the dynamic to reach a stable neural adaptation: to human voice adaptation would require more repetitions for the stabilization of P1 amplitude compared to their equivalent non-vocal sounds [71]. Another study did not draw the same conclusion with no RP for vocal sounds by only compared a few number of repetitions [73]. Using a roving paradigm with vocalizations varying in prosody, an enhanced effect of positive emotional content on the RP compared to angry and neutral vocalizations was observed [72]. Neural adaptation occurs in typical adults for sounds of different natures, but the natures of the sound could influence the pattern to reach a stable adaptation.

In fMRI studies, RS results in a decrease in brain activity with an increase in the number of repetitions of a standard and is referred to as fMRI adaptation [25]. To investigate fMRI adaptation, some studies have used pairs of images, with some of the pairs composed of the same two images and some consisting of two different images [74] and other studies have used classical oddball paradigms as in EEG [75,76,77]. In 2006, Sayres and Grill-Spector studied the implication of the duration of repetition of the same images [58]. In case of short presentation, the RS was significant, but with a smaller amplitude than for long presentations, suggesting as in other studies the involvement of attention in the adaptation process [24,78,79].

As in EEG, several fMRI studies have been conducted on deviance detection, showing an increase in the cerebral activity in response to the deviant compared to the standard stimulus [75]. Using oddball paradigms, a difference in brain activation in response to a deviant and to a standard stimulus was observed, that could be assimilated to the MMN and P3a (i.e., an index of attention orientation) in EEG studies [75,76,77,80,81,82]. In these studies, beside the expected Superior Temporal Gyrus (STG) activation, an involvement of the Inferior Frontal Gyrus (IFG) has repeatedly been reported [75,76,80,81,82].

More recently, studies using roving protocol have also been done in fMRI to study deviance detection, regularity encoding and prediction. Using a roving protocol with nine pure tones and four train lengths (4, 12, 24 and 36 repetitions) to localize the effect of adaptation, a variation in the intensity of the response to a deviant tone according the number of standards previously presented has been showed, which is consistent with prediction theory, and a dissociation between the regularity encoding and deviance detection in cortical subfields has been demonstrated [83].

### 2.3. Repetition Enhancement (RE)

The RE phenomenon has not been as well studied and described as RS but it is assumed that it corresponds to the expectation of stimulus appearance. It thus rather reflects prediction, i.e., the ability to anticipate a stimulus on the basis of previous experience [84]. RE is defined as an increase in the neural response with an increase in the number of repetitions [6,85]. Some studies observed RE with repetition, instead of RS, but only in response to degraded stimuli and concluded that RE could be a consequence of lack of access to memory representations through poor stimuli quality, preventing attenuation through the increasing perceptual performance. For example, Turk-Browne et al. [74] found RS when a scene with high visibility was repeated but reported RE when the same low-visibility scene was repeated. Thus, the nature of the stimulus could impact on the presence of either RS or RE. The difference could also reflect the features of the repeated stimulus, with RS related to the form and RE related to the size of the presented object in the same subjects [86].

Several models have proposed explanation for RE [6], among which are the accumulation model [27] and the novel network formation [87].

(1) The accumulation model proposes that the cumulative effect of repeated exposures leads to an enhanced or amplified response to the stimulus compared to its initial presentation, especially in case of qualitatively degraded stimulus (e.g., with low visibility) [27,87].

(2) In the novel network formation model, the presentation of a new stimulus leads to the creation of a new neural network coding for that stimulus. RE would thus reflect the creation of a new representation [6].

Studies intend to determine the difference between RE and RS in the brain regions implicated and in the timing of the setting up, by manipulating the nature of the stimulus or the paradigm. Comparing predictability of conditions, the RP occurs earlier in the more predictable condition, which is consistent with the idea of the novel network formation for RE [5]. In roving paradigms, RS was observed early with a decrease in N100 amplitude in response to pure sounds and then the RE occurs with an increase in P2 amplitude [5].

Neural adaptation has also been studied with magnetoencephalography (MEG), in visual [88] and auditory [67,89] modalities. Trying to localize the source of RS and RE, Recasens et al. demonstrated that the RS was generated in the Superior Temporal Gyrus (STG), the Middle Temporal Gyrus (MTG) and in insular regions whereas the RE sources were located in supratemporal and non-auditory regions (anterior region of the Insula and the Rolandic operculum) [67]. At the temporal level the two phenomena display different dynamic. Indeed, the RS has been identified as an early component (modulation of the early N1m, approximately at 90–150 ms) and the RE as a later component (sustained field, approximately at 230–270 ms) [67], confirming results of Costa-Faidella [5]. Moreover, these two components occur to have different implications. RS would correspond to the phenomenon of adaptation, whereas RE would rather correspond to anticipation of an expected event. Additionally, the dissociation between RS and RE has also been raised in fMRI studies demonstrating activation of different brain areas corresponding to the encoding of a new representation, and of brain regions involved in the retrieval of information [83,90,91]. Finally, the nature of the task and the required engagement of selective attention might influence the RS/RE balance [92]. Considering this modulation, RE would reflect selective attention in case of active oddball for example, whereas RS would be more likely observed in passive paradigms.

### 2.4. RS and RE Explained by Predictive Coding

Another model that can explain RS and RE is the predictive coding model [93]. The predictive coding hypothesis is a perceptual inference hypothesis describing the brain as a hierarchically organized cortical system that constantly attempts to anticipate future events. For this, the brain would constantly learn the regularities in the sensory environment to build predictions about future sensory inputs. Comparisons between information provided by sensory inputs (bottom-up) and generated predictions (top-down) of futures sensory inputs are thus performed at each level of the system [93,94,95]. The predictive coding theory takes up the main concept of the Bayes’ theory in which the percept (*posterior*) is based on belief and knowledge (*prior*) and on sensory input (*likelihood*). According to the strength of the *prior* and of the *likelihood*, the accuracy of the percept will be modified.

According to this hypothesis, perception will be biased by the quality of sensory inputs and predictions. Indeed, the stronger these two parameters are, the stronger will be their influence on perception. In a stable environment, the brain can extract strong regularities, which results in a robust prediction and thus, to a heavy influence of this parameter on perception. However, in an unstable environment, extraction of regularities will become more difficult, resulting in low predictions and thus in a weak influence of this parameter on perception. In parallel, if the sensory input comes from a clear environment, the precision of the incoming information will be high and thus the influence on perception would be stronger. However, if the sensory input comes from a noisy environment, the precision of the information contained will be low and thus the influence of this parameter will be weak [96,97].

**Figure 3:**
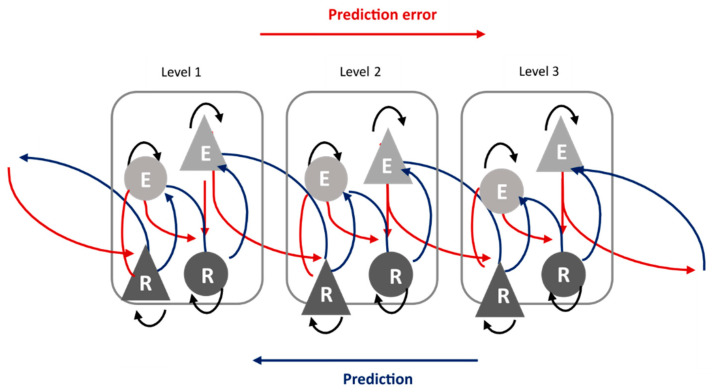
Predictive coding. In light grey: error unit; in dark grey: state unit. Triangle: pyramidal cells. Circle: bouquet cells. Red arrow: prediction error. Blue arrow: prediction. The levels correspond to hierarchical levels of cortical layers (adapted from “Attention, uncertainty, and free energy”, Frontiers in Human Neuroscience, 4, 215, Feldman and Friston (2010) [100]).

Moreover, sometimes sensory input and prediction are incongruent, resulting in the generation of a prediction error that leads to updating of the prediction, depending on the precision of this prediction error. Indeed, through the precision of the prediction error it can be determined if the system needs an update or if the prediction error is merely the result of low-quality sensory inputs or prediction, in which case it can be ignored [98,99]. The stronger the prediction (reflecting a strong regularity) is, the higher the precision of the prediction error will be, inducing prediction updating, which is, in turn, transmitted to lower areas [98,100] (Figure 3).

According to the predictive coding framework, RS, which reflects regularity encoding, would index a decrease in demand that occurs when expected and observed sensory information coincide (lower prediction error) and would thus reflect the increasing precision of the prediction [93]. Conversely, RE would index an increase in the prediction strength when expected and observed sensory information are the same [3,93,99]. MMN, previously described as an electrophysiological index of change detection, is considered to be a marker of prediction error and of updating of the prediction [101,102,103].

To conclude, the different explanations and models for adaptation and prediction to a repeated stimulus are summarized in Table 1.

Before discussing the possible implication of these neurophysiological processes in neurodevelopmental and psychiatric disorders, we summarize current knowledge regarding habituation, neural adaptation (RS and RE) and prediction in visual and auditory modalities with different methods (EEG, MEG, fMRI) through typical development.

## 3. Adaptation and Prediction through Typical Development

### 3.1. Behavioral Habituation and Familiarization

In children, habituation is an essential process for the creation of routine in infancy that improve health and wellbeing in families [104,105]. Several studies have shown that habituation is already present from infants (3 to 6 months old) [106,107], children (8 to 9 years) [108] to adults [15]. Some of these studies used fixation time, also referred as familiarization paradigm, with for example a visual pairs comparison paradigm, consisting of the presentation of pairs of similar and different images [109]. With this type of paradigm, it has been possible to observe preference for novelty, reflected by an increase in fixation time for a novel stimulus relative to the previously repeated stimulus [107,110,111]. However, using this procedure it makes it unclear whether the observed novelty preference indirectly reflects habituation, or not.

Nevertheless, some studies in infants using familiarization paradigms (in which an initial habituation phase composed of a repeated presentation of a stimulus is followed by a test phase with the presentation of a new stimulus [112]) have shown no preference for novelty but a preference for familiarity during the test phase, reflected by an increase in looking toward the familiar stimulus [113]. This result would be dependent on duration of habituation phase. Indeed, if a familiar stimulus has not been well encoded and represented, then it will be preferred during the test phase. The quality of the internal representation of a repeated stimulus, the complexity of the task and the age of the participants, would therefore influence the number of repetitions needed to reach a complete habituation [74,107,113]. Difference in habituation between adults and children could be the consequence of the effect of familiarity with the sentences used in this study [114]. The complexity of the stimulus to be encoded could also have an impact on the observed response [115]. In keeping with the idea that the RE is in place during the familiarization phase with a stimulation, in children a required number of repetitions was mandatory to eventually model a robust representation of it and produce a decrease in response, neural adaptation [63]. The familiarization phase with a new stimulus may be associated with RE and then the recognition of the familiar stimulus may be related to RS.

### 3.2. Neural Adaptation in Typical Development

In children, although behavioral habituation is present and efficient since the first months of life [106] and a need of routine is observed early in development [104,105], the underlying neural adaptation remains poorly explored. A description of the electrophysiology studies in this population is proposed in the current section.

There is no consensus on neural adaptation in children. Martineau et al. [116] observed RS in response to pure tones in children in an oddball. However, more recently, in a study comparing neural adaptation to electronic and human sounds, a decrease in the P1 amplitude was observed corresponding to RE, but only two repetitions were compared, suggesting that adaptation could take longer to implement [57]. Nevertheless, the presence of RE in children’s response is consistent with the idea presented earlier that RE instigates formation of the memory trace and after it is well established, RS is observed. These observations make sense in the predictive coding framework, in which RE could reflect prediction after a first step corresponding to familiarization [117].

To our knowledge, no studies have focused on adaptation in children using fMRI. Studies did not yet focus on the process of neural adaptation in response to different category of sounds. Additionally, no study using a properly designed paradigm to measure cortical adaptation in children has been conducted. The developmental trajectory of the ability of neural adaptation thus remains to be characterized.

The studies on neural adaptation in typically developing children and adults are summarized in Table 2. 

### 3.3. Prediction in Typical Development

Prediction in childhood and during development has not been as widely studied as in adulthood in the framework of the predictive coding theory. To obtain an idea of the prediction processes in this context during typical development, we will focus on the study of the prediction error electrophysiological correlate, i.e., the MMN [101,102,103]. Automatic detection of deviancy in a repetitive sequence has been studied in newborns [123,124], even in pre terms [125], in toddlers [126] and in children [127,128]. In these studies, a response to change has been observed in response to pitch or phonetic changes [123,124]. Additionally, the brain sources of this mismatch response reflecting the prediction appears to be different between 4 and 6 years old showing that the predictive brain is maturing during this time period [126]. In older children a mismatch response close to that of adults has been observed, with classic age-related differences in amplitudes and/or latencies [126,127]. However, although the characteristics of the response to sounds changes are not yet mature, the organization of the cortical response is already functional at the age of five [126,127,128]. The presence of prediction error since birth thus demonstrates that predictions are present early and effectively very early in development.

Additionally, as previously presented, RE is observed in children in familiarization and learning phases [63,117], which could reflect prediction and anticipation.

## 4. Adaptation and Prediction Are Altered in Neurodevelopmental and Psychiatric Disorders

What is known about adaptation and prediction in autism spectrum disorder (ASD), schizophrenia and attention-deficit/hyperactivity disorder (ADHD)? In these neurodevelopmental and psychiatric disorders, automatic detection of change has been widely studied through the measure of the MMN. This is true for ASD [129,130,131,132,133,134,135], for ADHD [136,137,138,139] and for schizophrenia [4,140,141,142,143]. In most of these studies, discrepancies in MMN amplitude and/or latency have been demonstrated in the clinical group compared to typically developing, but description of the underlying adaptation process is lacking. From a predictive coding point of view, description and thorough investigation of the prior adaptation process is essential to draw conclusions about difficulties in detection of change. As previously indicated, deficits in deviance detection could be explained by difficulties in underlying neural adaptation; understanding of these two phenomena in neurodevelopmental disorders is therefore important. In these disorders, in which difficulties in adaptation, prediction, and reaction to change have been demonstrated as a part of the symptomatology, a comprehension of the underlying neurophysiology is essential.

### 4.1. Autism Spectrum Disorder (ASD)

ASD is a neurodevelopmental disorder characterized by both social impairments and restrictive, repetitive interests reflecting a need of immutability [144]. One of the main characteristics of the ASD symptomatology is therefore resistance to change [145,146,147]. Two mechanisms could be involved: an habituation deficit [131] and/or an atypical change detection [133,134]. These adaptative difficulties in autistic people could be a consequence of their difficulties in predicting future events [148]. This idea has been developed in the theories of the Bayesian brain and predictive coding theory of ASD that postulate difficulties in predicting future items and in continually updating internal representations based on what has previously occurred [11,149,150]. According to Pellicano and Burr [11], the prior beliefs of autistic individuals are very different from those of a non-autistic group.

In autistic children a need for repetition and routine is observed, and has a positive influence on children leading to a facilitated adaptation [110,151]. Considering behavioral habituation, a reduction in response to repeated stimuli such as pure tones or vestibular stimulation has been observed in autistic children compared to neurotypical children [9,152]. This reduction of habituation could lead, as previously discussed, to resistance to change and could be the consequence of atypical neural adaptation. In 1992, Martineau et al. showed a lack of neural adaptation to pure tones through electrophysiological measurements [116]. Other electrophysiological studies have reached similar conclusions with no adaptation or less adaptation in autistic patients than in typically developing children [57,153,154,155]. However, all these studies draw conclusions on neural adaptation via oddball paradigms that are not specifically designed to study this process and by comparing only a restricted number of repetitions [153]. Not much research has used roving paradigms to study the implementation of the encoding of regularity and its associated cue, the RP, to determine whether it is affected in autism. Font-Alaminos et al. [156], using a roving paradigm to study the subcortical adaptation in autism, observed an increase in the amplitude of the frequency-following response (FFR), corresponding to RE. This is consistent with the idea of a neural adaptation deficit in individuals with ASD who do not adapt to a repeated stimulus but remain in a familiarization phase. Same conclusion has been reached by Latinus et al. [157] in an fMRI on cognitive flexibility. The results of EEG studies on neural adaptation are reported in Table 3.

In ASD, a disruption of the Excitatory/Inhibitory (E/I) balance in brain has been hypothesized [158]. In view of the above presented results in animals about the implication of the GABA_A_-mediated inhibition in SSA, the possible imbalance of the E/I in autism [159,160] could partly explain the differences observed in neural adaptation. The level of GABA/Glu also correlates with the ability to make correct prediction in the framework of Bayesian learning, especially in a frontal region in which autistic adults have lower levels of glutamate [161].

**Table 3:** Neural adaptation in autism. SOA: Stimulus Onset Asynchrony.

Study	Cortical/Subcortical	Modality	Population	Paradigm	Protocol	Stimuli Duration and SOA (ms)	Results
Martineau et al., 1992 [116]	Cortical	Auditory (pure tones)	30 children 3–11 years	Oddball	1 sequence of 60 tones	Stimuli: 100	▪ No decrease in the AER with the increase in repetitions
Guiraud et al., 2011 [155]	Cortical	Auditory (Pure tones)	35 infants with high risk for ASD and 21 infants with low risk for ASD 9 months old	Oddball	1 sequence (deviant always followed by two standards)	Stimuli: 100 SOA: 800	High risk: less marked ↘ (less habituation) and less ↗ response to deviant (reduced discrimination) Low risk: ▪ ↘ in P150 amplitude with standards repetition (habituation) ▪ ↗ in response amplitude to deviant (discrimination) High risk: ▪ No ↘ in P150 amplitude with standards repetition ▪ No ↗ in response amplitude to deviant
Gonzalez-Gadea et al., 2015 [154]	Cortical	Auditory (pure tones)	16 children 8–15 years	Oddball	2 blocks of 220 sequences, 3 types of sequence: ▪ Standard: 5 repetitions of the same tone ▪ Expected deviant: repetition of 4 identical tones, the fifth deviant tone is monaural Unexpected deviant: repetition of 4 identical tones, the fifth, deviant, tone is interaural	Stimuli: 50 SOA: 200 Inter sequence interval: between 700 and 1000	▪ No difference in MMN amplitude compared to typically developing participants ▪ P3 larger for expected deviant compared to standard ▪ No difference in P3 in unexpected deviant compared to typically developing participants ▪ Higher P3 amplitude in expected than in unexpected condition
Kolesnik et al., 2019 [162]	Cortical	Auditory (standard: pure tones Deviants: 1 white noise and 1 pure tone)	116 children with high risk of autism (9.03 +/− 1.1 m—39.05 +/− 3.47 m)	Oddball	1 sequence with standard and deviants (paradigm designed by Guiraud et al., 2011) [155] Measure the standard response after 1, 2 or 3 presentations	Stimuli: 100 Inter-trial interval: 700	▪ Reduced repetition suppression ▪ Increased phase locking
Font-Alaminos et al., 2020 [156]	Subcortical	Auditory (pure tones)	17 children 9.1 +/− 1.7 years	Roving (8, 10 and 12 repetitions)	1 sequence composed of 9 blocks, each block is composed of 30 trains of either 8, 10 and 12 repetitions	Stimuli: 100 SOA: 333	▪ ↗ FFR amplitude with repetition compared to typically developing participants
Ruiz-Martínez et al., 2020 [57]	Cortical	Auditory (Electronic and human sounds)	16 ASD children 7–10 years	Oddball	8 blocks (4/sound type) (deviant separated by at least 2 standards; each block begins with 10 standard)	Stimuli: 85 SOA: 685–885	▪ Reduced ↘ P1 response (reduced habituation) ▪ Reduced MMN amplitude (reduced discrimination)
Jamal et al., 2020 [121]	Cortical	Auditory (pure tones) and visual (radial checkerboard)	13 children 7.4–12.8 years	Sequences of repeated stimulus	2 sequences (1 visual and 1 auditory) with 300 repetitions of the same stimulus	SOA: 1,116 Stimuli duration: 116	▪ Reduced adaptation for both type of stimulus ▪ increase in the amplitude of the response
Cary et al., 2023 [153]	Cortical	Auditory (pure tones)	13 children 12.81 +/− 2.63 years	Oddball	1 sequence of 1000 trials (80% standard)	SOA: 600 Stimuli duration: 360	▪ Reduced P1 adaptation (between the first and the second standard) ▪ No difference between MMN amplitude with typically developing participants

Atypicalities reported in adaptation could reflect (or presume) the prediction deficit observed in autism and therefore hypersensitivity to the environment and intolerance to change [148].

In the predictive coding framework in ASD, the difficulties observed in response to deviance could reflect the lack of power of *priors* [11] or impaired encoding of sensory information [149,163,164]. Destabilization of the balance between the weight of sensory inputs and the weight of priors would lead to a high rates of prediction error and thus to impairment in deviance detection [165]. In autism there is a reduced ability to predict events and this could lead to deficits in understanding surroundings, which could explain difficulties in coping and may explain the emphasis on sameness, sensory hypersensitivities, difficulties in interacting with dynamic objects as well as difficulties in social cognition [166]. These different theories and assumptions were presented in a recent literature review on prediction in autistic people [167]. However, they do not cover all the major symptoms of autism, such as hyposensitivity, which would benefit further exploration and refined conceptualization. Randeniya et al. suggested that the processes of sensory learning and adaptation are important to interpret prediction error atypicalities in autism [168] and should also be further investigated.

Neural adaptation deficits, manifest in EEG and in fMRI, appear to be correlated with behavioral symptoms in ASD. High social impairment and sensory processing difficulties are indeed associated with reduced brain adaptation [121,169]. Differences related to the nature of the stimulus to be encoded have also been observed, with reduced fMRI adaptation for faces, but not for objects, in autistic adults, associated with challenges in social communication [170]. In response to sounds, fMRI adaptation in autistic adults also appears reduced in comparison to a group of typically developing adults [171].

In conclusion, in ASD the process of habituation/adaptation appeared to be impaired, and this may partially explain hypersensitivity to change, and difficulties observed in detecting change. Further studies of these phenomena in autism, with more ecological stimuli, social for example, could make it possible to determine the possible implication of the context, and of the nature of the repeated stimuli in resistance to change. A few studies have compared adaptation to social and non-social information in autism and have revealed differences according to the nature of the stimuli, with less (or an absence of) adaptation for social stimuli in comparison to non-social stimuli [10,172]. In other words, reduced sensory adaptation in autism could contribute to hypersensitivities but also to difficulties in social communication and adaptation to the environment.

### 4.2. Schizophrenia

Schizophrenia is a psychiatric disorder characterized by several symptoms such as disturbances in thought, perception and behavior, according to the DSM-5 [144]. Habituation also seems to be impacted in this disorder. It has been proposed that in schizophrenia there is an atypical interpretation of incoming input due to inappropriate creation and use of stored regularities [173,174,175]. We propose here a review of the current state of knowledge about habituation and adaptation in this disorder.

Regarding behavioral habituation in schizophrenia, impairment has been observed, with less marked habituation to repeated events (i.e., sounds, images) compared to typically developing [8,176,177]. The habituation deficit in schizophrenia may be related to the memory impairment observed in this disorder. fMRI studies have shown that the lack of habituation was associated with a lack of activation of the hippocampus, a brain region involved in memory, in response to repeated stimuli [178,179].

There is no consensus on neural adaptation in schizophrenia based on EEG studies. Atypical deviance detection has repeatedly been observed in schizophrenia patients compared to neurotypical, with reduced MMN [4,70,71,180,181,182]. Studies using the roving paradigm to explore regularity encoding and neural adaptation by measuring the RP have reached divergent conclusions. In some studies, a reduced RP was observed in schizophrenic patients compared to typically developing participants [4,121], while another study reported no difference in the RP between patients and neurotypical [70]. In an oddball study, RS on several electrophysiological components (N100, P50 and P2) was observed [181]. As presented above, neural adaptation can be established in patients with schizophrenia, but not as efficiently as in controls, which could partially explain the deficit in habituation observed in schizophrenia. A summary of these different electrophysiological studies and their conclusions are reported in Table 4.

Moreover, as in ASD, it has been argued that the predictive coding theory accounts for the underlying mechanisms involved in this altered MMN [142] since there is a reduced effect of the repetition on the amplitude of the MMN reflecting an impairment in prediction [185].

It remains complicated to draw conclusions regarding neural adaptation in schizophrenia. Indeed, there is still no consensus on fMRI adaptation in these patients. Some studies have found similar fMRI adaptation for repeated faces between typically developing participants and schizophrenic patients in specialized brain areas such as the fusiform face area (FFA) and occipital face area (OFA) [186,187]. However, in Williams et al.’s study [187], no RS was found in less specialized brain regions, with no decrease in the response amplitude with repetition in the primary visual cortex and in the hippocampus in comparison to neurotypical. Authors conclude that adaptation appears to be preserved in ultra-specialized brain areas in schizophrenia, but not in the more general areas responsible for encoding basic features of visual stimuli [187]. Furthermore, another study found smaller fMRI adaptation in patients with schizophrenia compared to typically developing participants in response to pictures of objects in the lateral occipital cortex (LOC), the brain area responsive to the category of visual stimuli [188]. This study represents a complementary finding to what was previously presented; adaptation at the brain level appears to be possible in specialized areas but appears to be impaired in more generic brain areas responsible for visual processing, reflecting alteration in the whole brain response but not in ultra-specialized areas in schizophrenia.

To summarize, in schizophrenia the habituation difficulties observed in behavior are not necessarily related to basic deficits in neural adaptation as observed in EEG and/or in fMRI. New theories about predictive coding in schizophrenia may help to draw conclusions about the mechanisms involved in these difficulties.

### 4.3. Attention-Deficit/Hyperactivity Disorder (ADHD)

According to the DSM-5, ADHD is a neurodevelopmental disorder characterized by a persistent pattern of inattention and/or hyperactivity-impulsivity [144]. The deficits in attention could lead to adaptation and habituation impairment, because of difficulties in regularity encoding due to early inattention.

At the behavioral level, habituation has been shown to be impaired in ADHD in visual tasks [189,190]. However, the nature of the measure has an impact on the habituation ability, with some skin conductance studies revealing enhanced and accelerated habituation to sounds in startle reflex paradigms compared to typically developing adults [191,192]. This quicker habituation would induce a less efficient reinforcement of the memory trace and consequently a less sustainable habituation [193].

In ADHD there is no study focusing on neural regularity encoding. The possible adaptation and habituation deficit is mostly studied by observing changes in attention, through the P3, an index of attention orientation. P3 has been shown to be altered in ADHD, with no difference in amplitude between expected and unexpected conditions for example [154]. This attentional switch deficit could lead to an adaptation deficit. Indeed, in case of impairment in the regularity encoding and in adaptation (less expectation) to standard stimuli, the detection of change and novelty involved in attention switching become more difficult. Predictive coding theory could provide one explanation: in ADHD the observed defect in P3 could be related to too much involvement of sensory inputs at the expense of predictions [154,194].

Other studies have found no difference in MMN amplitude between neurotypical and individuals with ADHD [138,154], indicating that even if attentional switching is atypical in ADHD, deviance detection does not seem to be impaired. However, a meta-analysis on MMN in ADHD patients revealed that in these patients the amplitude of this index is reduced compared to typically developing children [195]. As in ASD and schizophrenia, change detection appears to be impaired. The joint action of change detection and attentional switching deficits could therefore be indicative of a neural adaptation deficit, but to our knowledge no study has yet addressed this cascade of processes in ADHD.

## 5. Future Directions

Future study of neural adaptation throughout typical development and in neurodevelopmental disorders would be interesting, especially in response to more complex stimuli than pure tones or basic shapes. Indeed, the study of the combination of social cues on adaptation, especially in ASD, could allow for better understanding of the effects that have already been observed regarding change detection, and could provide a link with the symptomatic dyad, sameness and aloneness, initially described by Kanner [196] and Soukhareva [197]. Additionally, the study of neural adaptation in neurodevelopmental disorders will help answer the research question of whether there is a problem in change detection itself or a problem in subjacent adaptation that is behind the MMN atypicalities. The response to this question would have a major impact on targeted behavioral intervention in these populations.

## 6. Conclusions

In summary, habituation and adaptation are important phenomena in the encoding of a steady context and in the detection of changes in the environment. These mechanisms are studied at different levels, but recently a more adapted electrophysiological cue has been measured: repetition positivity, which reflects regularity encoding and therefore neural adaptation in the framework of the roving paradigm. In the neurodevelopmental disorders discussed in this review, behavioral habituation seems to be impaired, but specifically designed studies of neural adaptation are not yet widespread. In future studies, it would be relevant to investigate these adaptation mechanisms using more complex stimuli that may contain social cues, for example in order to study their involvement in other components of the symptomatology of neurodevelopmental disorders, such as socio-communicative difficulties.

## Figures and Tables

**Table 1:** Summary of the processes underlying repetition suppression (RS) and repetition enhancement (RE) according to the different theories/models.

	Repetition Suppression	Repetition Enhancement
	Decrease in neural activity to repeated presentation of the same stimulus **→ Reflects novelty preference**	Increase in the response as the number of repetitions of a stimulus increases. More marked for degraded stimuli, reflecting missing access to memory representation and construction of this representation. **→ Reflects familiarity preference**
**Grill-Spector et al.’s Models** Grill-Spector et al., 2006 [23]	**The fatigue model**: decrease in the amplitude of firing of neurons responding to the stimulus, proportional to the initial response	**The novel network formation**: presentation of a novel stimulus that is learned and its representation is established with the creation of a new neural network coded for this stimulus.
	**The sharpening model**: decrease in the number of neurons that respond to the stimulus with repeated presentation	
**The facilitation (or accumulation) model** James and Gauthier, 2006 [27]	Faster processing of the stimulus by the neurons involved, resulting in shorter latency or reaction time	Repeated exposure to stimulus with low visibility generally leads to increased perceptual performance.
**Predictive coding** Friston, 2005 [93]	Regularity encoding, displaying a decrease in the demand that occurs when expected and observed sensory information coincide. Reflects the increasing precision of prediction, the correct prediction of the upcoming stimulus	Increase in the prediction weight when expected and observed sensory information are the same.

**Table 2:** Neural adaptation in typically developing adults and children. SOA: Stimulus Onset Asynchrony.

Study	Cortical/Subcortical	Modality	Population	Paradigm	Protocol	Stimuli Duration and SOA (ms)	Results
Baldeweg et al., 2004 [4]	Cortical	Auditory (pure tones)	20 adults	Roving (2, 6, 18 and 38 repetitions)	1 sequence	Stimuli: 25 for standard and 50 for deviant	▪ ↗MMN with ↗ number of repetitions ▪ Detection of RP
Haenschel et al., 2005 [66]	Cortical	Auditory (pure tones)	40 adults	Roving (trains of 2, 6 and 36 repetitions)	2 blocks with passive listening 2 blocks with active discrimination	Stimuli: 200 SOA: 500	▪ ↗MMN with ↗ number of repetitions ▪ Detection of RP (36—2 repetitions)
Ylinen and Huotilainen, 2007 [73]	Cortical	Auditory (synthetized vowels and vowels-like equivalents)	9 adults	Roving (3–4 repetitions 5–6 repetitions)	1 sequence of familiar stimuli (vowels) 1 sequence of unfamiliar stimuli (vowels-like)	Stimuli: 400 SOA: 700	▪ Ø RP for both conditions ▪ ↗ N1 amplitude for familiar stimuli only
Garrido et al., 2009 [118]	Cortical	Auditory (pure tones)	10 adults	Roving	1 sequence	Stimuli: 70 SOA: 570	▪ ↗MMN with ↗ number of repetitions
Costa-Faidella et al., 2011 [56]	Cortical	Auditory (pure tones)	17 adults	Roving (trains of 3, 6 and 12 repetitions)	Predictable condition and unpredictable condition	Stimuli: 50 SOA: 708 (predictable) or 364–1062 (unpredictable)	▪ Detection of RP for predictable condition Only later part of RP (>200 ms) observed in unpredictable condition
Costa-Faidella et al., 2011 [5]	Cortical	Auditory (pure tones)	20 adults	Oddball (2, 6 and 12 repetitions)	1 sequence composed of 2 runs. Run 1: S1 repeated 2, 6 or 12 times, followed by deviant S2. Run 2: S2 repeated 2, 6 or 12 times, followed by deviant S1	Stimuli: 40	▪ ↗ P2 amplitude with repetition ▪ ↗ MMN amplitude with repetition ▪ ↘ N100 amplitude with repetition
Cooper et al., 2013 [68]	Cortical	Auditory (pure tones)	24 adults	Roving and oddball (4, 8 or 16 standards)	1 roving sequence 1 oddball sequence	Stimuli: 50 for standard and 100 for deviant	▪ RP observed only for roving condition ▪ Ø increase in MMN amplitude with repetition for both condition
Recasens et al., 2015 [67]	Cortical	Auditory (pure tones)	13 adults	Roving (3, 12 or 24 repetitions)	2 runs: 198 trains of each length	Stimuli: 50 SOA: 500	▪ Early N1m: RS from initial to late repetitions in supratemporal regions and in non-auditory regions as the precuneus ▪ Late SF interval: RE from initial to late repetitions in the HG, the STG, the MTG and insular regions
Gorina-Careta et al., 2016 [119]	Subcortical	Auditory (consonant-vowel)	30 adults	Sequence of repeated stimuli	Predictable timing condition and unpredictable timing condition (8 blocks of 1001 repetitions per condition)	Stimuli: 170 SOA: 366 (predictable) or 183– 549 (unpredictable)	▪ ↘ FFR with repetition for both conditions ▪ stronger ↘ FFR for predictable condition
Pinheiro et al., 2017 [72]	Cortical	Auditory (human vocalizations)	23 adults	Oddball	4 blocks of 1050 standards and 150 deviants. Three different stimuli, standard or deviant, depending on the block: neutral, angry and happy	Stimuli 700 SOA: 1200	▪ RP amplitude higher for happy vocalizations than angry and neutral and increased with repetitions
McCleery et al., 2019 [70]	Cortical	Auditory (pure tones)	29 adults	Roving (3, 8 or 33 repetitions)	1 sequence composed of 5 blocks at two different times (2 weeks apart) Stimuli vary in pitch + duration	Stimuli: 50 or 100	▪ ↗ MMN with repetition ▪ ↗ amplitude of the last standard of a train (3, 8, 33) with repetition (called RP)
Fryer et al., 2020 [120]	Cortical	Auditory (pure tones)	241 adults	Oddball	3 blocks consisting of a standard (85%), a deviant in duration (5%), a deviant in frequency (5%), and a deviant in duration and in frequency (5%)	SOA: 500 Stimuli standard in duration: 50 Deviant in duration: 100	▪ RP amplitude linearly increased with the repetition of the standard
Jamal et al., 2020 [121]	Cortical	Auditory (pure tones) and visual (radial checkerboard)	22 children 7.1–12.8 years	Sequences of repeated stimulus	2 sequences (1 visual and 1 auditive) with 300 repetitions of the same stimulus	SOA: 1116 Stimuli duration: 116	▪ Decrease in the ERP amplitude with repetitions for both type of stimuli
Ruiz-Martínez et al., 2020 [57]	Cortical	Auditory (Electronic and human sounds)	15 children 5–11 years	Oddball	8 blocks (4/sound type) (deviant separated by at least 2 standards; each block begins with 10 standards)	Stimuli: 85 SOA: 685–885	▪ ↘ P1 response (only two repetitions tested)
Feuerriegel et al., 2021 [122]	Cortical	Visual (faces)	43 adults	Oddball	34 sequences: 6 faces presented, then oddball face identity (different identity than the base face). Blocks of 6 consecutive sequences were used with two oddball face identities possible, the proportion of both is known	Images were presented at a rate of 6 Hz	▪ Surprising compared to neutral: VMR amplitude is more negative in early and late time windows No VMR amplitude difference between expected and neutral conditions in early and late time windows
Heurteloup et al., 2022 [71]	Cortical	Auditory (complex tones and human voice)	20 adults (18 to 30 years)	Roving (4, 8 or 16 repetitions)	2 roving sequences: one for vocal sounds and one for complex non-vocal sounds	SOA: 646 Stimuli duration: 300	▪ RP for both categories of sounds ▪ Decrease in the ERP amplitude with repetitions for both type of stimuli, but faster for complex sounds compared to vocal sounds

**Table 4:** Neural adaptation in schizophrenia. SOA: Stimulus Onset Asynchrony.

Study	Cortical/Subcortical	Modality	Population	Paradigm	Protocol	Stimuli Duration and SOA (ms)	Results
Baldeweg et al., 2004 [4]	Cortical	Auditory (pure tones)	28 adults	Roving (2, 6, 18 and 38 repetitions)	1 sequence	Stimuli: 25 for standard and 50 for deviant	▪ Reduced MMN compared to typically developing participants ▪ Ø increase RP with repetition ▪ Reduced RP compared to controls
Rentzsch et al., 2015 [183]	Cortical	Auditory (pure tones and click sound)	25 adults	Oddball and paired click paradigm	Oddball: standard and deviant stimuli differed in frequency Click: pair of identical stimuli repeated	Oddball: Pure tone duration: 80 Inter-stimulus interval: between 350 and 650 ms Click sound: SOA: 500 and inter-trial interval of 3400	▪ Reduced MMN compared to typically developing participants ▪ No effect on RS for P50 ▪ N100 and P200 RS reduced
Coffman et al., 2017 [181]	Cortical	Auditory (pure tones)	26 adults	Oddball	2 tasks: ▪ RS task: 5 repeated similar tones MMN task: 5 repeated tones and pitch or duration mismatch	Stimuli: 50 SOA: 330 Inter-group: 750 Stimuli duration mismatch: 100	▪ RS in P50, N100 and P200 responses ▪ Reduced MMN compared to control
McCleery et al., 2019 [70]	Cortical	Auditory (pure tones)	43 adults	Roving (3, 8 or 33 repetitions)	1 sequence composed of 5 blocks at two different times (2 weeks apart). Pitch + duration stimuli variation	Stimuli: 50 or 100	▪ ↗ MMN with repetition but reduced MMN compared to controls ▪ ↗ amplitude of the last standard of a train (3, 8, 33) with repetition (called RP) as in typically developing participants
Fryer et al., 2020 [120]	Cortical	Auditory (pure tones)	54 adults with Psychosis Risk syndrome—Conversion (PRS-C)	Oddball	3 blocks consisting of a standard (85%), a deviant in duration (5%), a deviant in frequency (5%), and a deviant in duration and in frequency (5%)	SOA: 500 Stimuli standard in duration: 50 Deviant in duration: 100	▪ RP amplitude smaller than typically developing participants ▪ Effect of the position in the train especially in position 3, 8–10 and 11+ in the train
Koshiyama et al., 2020 [182]	Cortical	Auditory (pure tones)	25 adults	Oddball and many-standards paradigm	Oddball: 2 sequences ▪ Duration: 2 tones with different durations ▪ Frequency: 2 tones with different frequencies Many-standards paradigm: 2 sequences ▪ Duration: 10 tones with different durations ▪ Frequencies: 10 tones with different frequencies	Stimulus standard: 50 Oddball duration sequence → deviants 100 Many-standards paradigm: between 10 and 225	▪ Reduced MMN compared to typically developing participants ▪ No difference in adaptation between patients and typically developing participants ▪ Reduced deviant detection component of the MMN in patients
Mazer et al., 2021 [184]	Cortical	Auditory (bird song, voice and pure tone)	26 adults	Roving	7 repetitions for trains for bird song and voice—target tone appears between train	Complex sounds (bird songs and voice): 200 Inter-stimulus interval: 1000 Pure tone: 70	▪ No decrease in P2 amplitude for bird songs ▪ Decrease in P2 amplitude for human voice ▪ No difference in N1 habituation between patients and typically developing participants

## Data Availability

Not applicable.

## References

[1] Winkler I., Denham S.L., Nelken I. (2009). Modeling the Auditory Scene: Predictive Regularity Representations and Perceptual Objects. Trends Cogn. Sci..

[2] Andrews T.J., Ewbank M.P. (2004). Distinct Representations for Facial Identity and Changeable Aspects of Faces in the Human Temporal Lobe. Neuroimage.

[3] Summerfield C., Trittschuh E.H., Monti J.M., Mesulam M.-M., Egner T. (2008). Neural Repetition Suppression Reflects Fulfilled Perceptual Expectations. Nat. Neurosci..

[4] Baldeweg T., Klugman A., Gruzelier J., Hirsch S.R. (2004). Mismatch Negativity Potentials and Cognitive Impairment in Schizophrenia. Schizophr. Res..

[5] Costa-Faidella J., Grimm S., Slabu L., Díaz-Santaella F., Escera C. (2011). Multiple Time Scales of Adaptation in the Auditory System as Revealed by Human Evoked Potentials: Adaptation in the Human Auditory System. Psychophysiology.

[6] Segaert K., Weber K., de Lange F.P., Petersson K.M., Hagoort P. (2013). The Suppression of Repetition Enhancement: A Review of FMRI Studies. Neuropsychologia.

[7] Parras G.G., Nieto-Diego J., Carbajal G.V., Valdés-Baizabal C., Escera C., Malmierca M.S. (2017). Neurons along the Auditory Pathway Exhibit a Hierarchical Organization of Prediction Error. Nat. Commun..

[8] Avery S.N., Armstrong K., Blackford J.U., Woodward N.D., Cohen N., Heckers S. (2019). Impaired Relational Memory in the Early Stage of Psychosis. Schizophr. Res..

[9] Ramaswami M. (2014). Network Plasticity in Adaptive Filtering and Behavioral Habituation. Neuron.

[10] Ewbank M.P., Pell P.J., Powell T.E., von dem Hagen E.A.H., Baron-Cohen S., Calder A.J. (2017). Repetition Suppression and Memory for Faces Is Reduced in Adults with Autism Spectrum Conditions. Cereb. Cortex.

[11] Pellicano E., Burr D. (2012). When the World Becomes “Too Real”: A Bayesian Explanation of Autistic Perception. Trends Cogn. Sci..

[12] Groves P.M., Thompson R.F. (1970). Habituation: A Dual-Process Theory. Psychol. Rev..

[13] Thompson R.F., Spencer W.A. (1966). Habituation: A Model Phenomenon for the Study of Neuronal Substrates of Behavior. Psychol. Rev..

[14] Hepper P.G., Leader L.R. (1996). Fetal Habituation. Fet. Matern. Med. Rev..

[15] Rankin C.H., Abrams T., Barry R.J., Bhatnagar S., Clayton D.F., Colombo J., Coppola G., Geyer M.A., Glanzman D.L., Marsland S. (2009). Habituation Revisited: An Updated and Revised Description of the Behavioral Characteristics of Habituation. Neurobiol. Learn. Mem..

[16] Giles A.C., Rankin C.H. (2009). Behavioral and Genetic Characterization of Habituation Using Caenorhabditis Elegans. Neurobiol. Learn. Mem..

[17] Bronstein P.M., Neiman H., Wolkoff F.D., Levine M.J. (1974). The Development of Habituation in the Rat. Anim. Learn. Behav..

[18] Pinsker H., Kupfermann I., Castellucci V., Kandel E. (1970). Habituation and Dishabituation of the Gill-Withdrawal Reflex in Aplysia. Science.

[19] Bell R., Röer J.P., Dentale S., Buchner A. (2012). Habituation of the Irrelevant Sound Effect: Evidence for an Attentional Theory of Short-Term Memory Disruption. J. Exp. Psychol. Learn. Mem. Cogn..

[20] Ornitz E.M., Guthrie D. (1989). Long-Term Habituation and Sensitization of the Acoustic Startle Response in the Normal Adult Human. Psychophysiology.

[21] Pellegrino R., Sinding C., de Wijk R.A., Hummel T. (2017). Habituation and Adaptation to Odors in Humans. Physiol. Behav..

[22] Desimone R. (1996). Neural Mechanisms for Visual Memory and Their Role in Attention. Proc. Natl. Acad. Sci. USA.

[23] Grill-Spector K., Henson R., Martin A. (2006). Repetition and the Brain: Neural Models of Stimulus-Specific Effects. Trends Cogn. Sci..

[24] Avidan G., Hasson U., Hendler T., Zohary E., Malach R. (2002). Analysis of the Neuronal Selectivity Underlying Low FMRI Signals. Curr. Biol..

[25] Grill-Spector K., Malach R. (2001). FMR-Adaptation: A Tool for Studying the Functional Properties of Human Cortical Neurons. Acta Psychol..

[26] Wiggs C.L., Martin A. (1998). Properties and Mechanisms of Perceptual Priming. Curr. Opin. Neurobiol..

[27] James T.W., Gauthier I. (2006). Repetition-Induced Changes in BOLD Response Reflect Accumulation of Neural Activity. Hum. Brain Mapp..

[28] Ulanovsky N., Las L., Farkas D., Nelken I. (2004). Multiple time scales of adaptation in auditory cortex neurons. J. Neurosci..

[29] Baylis G.C., Rolls E.T. (1987). Responses of Neurons in the Inferior Temporal Cortex in Short Term and Serial Recognition Memory Tasks. Exp. Brain Res..

[30] De Baene W., Vogels R. (2010). Effects of Adaptation on the Stimulus Selectivity of Macaque Inferior Temporal Spiking Activity and Local Field Potentials. Cereb. Cortex.

[31] Dragoi V., Sharma J., Sur M. (2000). Adaptation-Induced Plasticity of Orientation Tuning in Adult Visual Cortex. Neuron.

[32] Kaliukhovich D.A., Vogels R. (2011). Stimulus Repetition Probability Does Not Affect Repetition Suppression in Macaque Inferior Temporal Cortex. Cereb. Cortex.

[33] Kaliukhovich D.A., Vogels R. (2012). Stimulus Repetition Affects Both Strength and Synchrony of Macaque Inferior Temporal Cortical Activity. J. Neurophysiol..

[34] Kaliukhovich D.A., Vogels R. (2014). Neurons in Macaque Inferior Temporal Cortex Show No Surprise Response to Deviants in Visual Oddball Sequences. J. Neurosci..

[35] Liu Y., Murray S.O., Jagadeesh B. (2009). Time Course and Stimulus Dependence of Repetition-Induced Response Suppression in Inferotemporal Cortex. J. Neurophysiol..

[36] Miller E.K., Li L., Desimone R. (1991). A neural mechanism for working and recognition memory in inferior temporal cortex. Science.

[37] Müller J.R., Metha A.B., Krauskopf J., Lennie P. (1999). Rapid Adaptation in Visual Cortex to the Structure of Images. Science.

[38] Ringo J.L. (1996). Stimulus Specific Adaptation in Inferior Temporal and Medial Temporal Cortex of the Monkey. Behav. Brain Res..

[39] Sawamura H., Orban G.A., Vogels R. (2006). Selectivity of Neuronal Adaptation Does Not Match Response Selectivity: A Single-Cell Study of the FMRI Adaptation Paradigm. Neuron.

[40] Vinken K., Vogels R. (2017). Adaptation Can Explain Evidence for Encoding of Probabilistic Information in Macaque Inferior Temporal Cortex. Curr. Biol..

[41] Kuravi P., Vogels R. (2018). GABAergic and Cholinergic Modulation of Repetition Suppression in Inferior Temporal Cortex. Sci. Rep..

[42] Ulanovsky N., Las L., Nelken I. (2003). Processing of Low-Probability Sounds by Cortical Neurons. Nat. Neurosci..

[43] Fishman Y.I., Steinschneider M. (2012). Searching for the Mismatch Negativity in Primary Auditory Cortex of the Awake Monkey: Deviance Detection or Stimulus Specific Adaptation?. J. Neurosci..

[44] Nelken I., Yaron A., Polterovich A., Hershenhoren I. (2013). Stimulus-Specific Adaptation beyond Pure Tones. Adv. Exp. Med. Biol..

[45] Taaseh N., Yaron A., Nelken I. (2011). Stimulus-Specific Adaptation and Deviance Detection in the Rat Auditory Cortex. PLoS ONE.

[46] von der Behrens W., Bäuerle P., Kössl M., Gaese B.H. (2009). Correlating Stimulus-Specific Adaptation of Cortical Neurons and Local Field Potentials in the Awake Rat. J. Neurosci..

[47] Yaron A., Hershenhoren I., Nelken I. (2012). Sensitivity to Complex Statistical Regularities in Rat Auditory Cortex. Neuron.

[48] Anderson L.A., Malmierca M.S. (2013). The Effect of Auditory Cortex Deactivation on Stimulus-Specific Adaptation in the Inferior Colliculus of the Rat. Eur. J. Neurosci..

[49] Ayala Y.A., Malmierca M.S. (2012). Stimulus-Specific Adaptation and Deviance Detection in the Inferior Colliculus. Front. Neural. Circuits.

[50] Pérez-González D., Malmierca M.S., Covey E. (2005). Novelty Detector Neurons in the Mammalian Auditory Midbrain. Eur. J. Neurosci..

[51] Pérez-González D., Malmierca M.S. (2012). Variability of the Time Course of Stimulus-Specific Adaptation in the Inferior Colliculus. Front. Neural. Circuits.

[52] Anderson L.A., Christianson G.B., Linden J.F. (2009). Stimulus-Specific Adaptation Occurs in the Auditory Thalamus. J. Neurosci..

[53] Antunes F.M., Nelken I., Covey E., Malmierca M.S. (2010). Stimulus-Specific Adaptation in the Auditory Thalamus of the Anesthetized Rat. PLoS ONE.

[54] Antunes F.M., Malmierca M.S. (2011). Effect of Auditory Cortex Deactivation on Stimulus-Specific Adaptation in the Medial Geniculate Body. J. Neurosci..

[55] Duque D., Malmierca M.S., Caspary D.M. (2014). Modulation of Stimulus-Specific Adaptation by GABA(A) Receptor Activation or Blockade in the Medial Geniculate Body of the Anaesthetized Rat. J. Physiol..

[56] Costa-Faidella J., Baldeweg T., Grimm S., Escera C. (2011). Interactions between “What” and “When” in the Auditory System: Temporal Predictability Enhances Repetition Suppression. J. Neurosci..

[57] Ruiz-Martínez F.J., Rodríguez-Martínez E.I., Wilson C.E., Yau S., Saldaña D., Gómez C.M. (2020). Impaired P1 Habituation and Mismatch Negativity in Children with Autism Spectrum Disorder. J. Autism Dev. Disord..

[58] Sayres R., Grill-Spector K. (2006). Object-Selective Cortex Exhibits Performance-Independent Repetition Suppression. J. Neurophysiol..

[59] Snyder K.A., Keil A. (2008). Repetition Suppression of Induced Gamma Activity Predicts Enhanced Orienting toward a Novel Stimulus in 6-Month-Old Infants. J. Cogn. Neurosci..

[60] Näätänen R., Gaillard A.W., Mäntysalo S. (1978). Early Selective-Attention Effect on Evoked Potential Reinterpreted. Acta Psychol..

[61] Näätänen R., Paavilainen P., Rinne T., Alho K. (2007). The Mismatch Negativity (MMN) in Basic Research of Central Auditory Processing: A Review. Clin. Neurophysiol..

[62] Winkler I., Schröger E., Cowan N. (2001). The Role of Large-Scale Memory Organization in the Mismatch Negativity Event-Related Brain Potential. J. Cogn. Neurosci..

[63] Grossmann T., Gliga T., Johnson M.H., Mareschal D. (2009). The Neural Basis of Perceptual Category Learning in Human Infants. J. Cogn. Neurosci..

[64] Baldeweg T., Williams J.D., Gruzelier J.H. (1999). Differential Changes in Frontal and Sub-Temporal Components of Mismatch Negativity. Int. J. Psychophysiol..

[65] Cowan N., Winkler I., Teder W., Naatanen R. (1993). Memory Prerequisites of Mismatch Negativity in the Auditory Event-Related Potential (ERP). J. Exp. Psychol. Learn. Mem. Cogn..

[66] Haenschel C., Vernon D.J., Dwivedi P., Gruzelier J.H., Baldeweg T. (2005). Event-Related Brain Potential Correlates of Human Auditory Sensory Memory-Trace Formation. J. Neurosci..

[67] Recasens M., Leung S., Grimm S., Nowak R., Escera C. (2015). Repetition Suppression and Repetition Enhancement Underlie Auditory Memory-Trace Formation in the Human Brain: An MEG Study. NeuroImage.

[68] Cooper R.J., Atkinson R.J., Clark R.A., Michie P.T. (2013). Event-Related Potentials Reveal Modelling of Auditory Repetition in the Brain. Int. J. Psychophysiol..

[69] Garrido M.I., Kilner J.M., Stephan K.E., Friston K.J. (2009). The Mismatch Negativity: A Review of Underlying Mechanisms. Clin. Neurophysiol..

[70] McCleery A., Mathalon D.H., Wynn J.K., Roach B.J., Hellemann G.S., Marder S.R., Green M.F. (2019). Parsing Components of Auditory Predictive Coding in Schizophrenia Using a Roving Standard Mismatch Negativity Paradigm. Psychol. Med..

[71] Heurteloup C., Merchie A., Roux S., Bonnet-Brilhault F., Escera C., Gomot M. (2022). Neural Repetition Suppression to Vocal and Non-Vocal Sounds. Cortex.

[72] Pinheiro A.P., Barros C., Vasconcelos M., Obermeier C., Kotz S.A. (2017). Is Laughter a Better Vocal Change Detector than a Growl?. Cortex.

[73] Ylinen S., Huotilainen M. (2007). Is There a Direct Neural Correlate for Memory-Trace Formation in Audition?. NeuroReport.

[74] Turk-Browne N., Yi D.-J., Leber A., Chun M. (2007). Visual Quality Determines the Direction of Neural Repetition Effects. Cereb. Cortex.

[75] Doeller C.F., Opitz B., Mecklinger A., Krick C., Reith W., Schröger E. (2003). Prefrontal Cortex Involvement in Preattentive Auditory Deviance Detection:: Neuroimaging and Electrophysiological Evidence. NeuroImage.

[76] Opitz B., Rinne T., Mecklinger A., von Cramon D.Y., Schröger E. (2002). Differential Contribution of Frontal and Temporal Cortices to Auditory Change Detection: FMRI and ERP Results. NeuroImage.

[77] Opitz B., Schröger E., Cramon D.Y.V. (2005). Sensory and Cognitive Mechanisms for Preattentive Change Detection in Auditory Cortex. Eur. J. Neurosci..

[78] Eger E., Henson R.N.A., Driver J., Dolan R.J. (2004). BOLD Repetition Decreases in Object-Responsive Ventral Visual Areas Depend on Spatial Attention. J. Neurophysiol..

[79] Murray S.O., Wojciulik E. (2003). Attention Increases Neural Selectivity in the Human Lateral Occipital Complex. Nat. Neurosci..

[80] Gomot M., Bernard F.A., Davis M.H., Belmonte M.K., Ashwin C., Bullmore E.T., Baron-Cohen S. (2006). Change Detection in Children with Autism: An Auditory Event-Related FMRI Study. NeuroImage.

[81] Sabri M., Kareken D.A., Dzemidzic M., Lowe M.J., Melara R.D. (2004). Neural Correlates of Auditory Sensory Memory and Automatic Change Detection. NeuroImage.

[82] Schall U., Johnston P., Todd J., Ward P.B., Michie P.T. (2003). Functional Neuroanatomy of Auditory Mismatch Processing: An Event-Related FMRI Study of Duration-Deviant Oddballs. NeuroImage.

[83] Cacciaglia R., Costa-Faidella J., Zarnowiec K., Grimm S., Escera C. (2019). Auditory Predictions Shape the Neural Rsponses to Stimulus Repetition and Sensory Change. Neuroimage.

[84] Sokolov E.N., Sokolov Y.N., Sokolov E.N., Waydenfeld S.W., Worters R., Clarke A.D.B. (1963). Perception and the Conditioned Reflex.

[85] Vogels R. (2016). Sources of Adaptation of Inferior Temporal Cortical Responses. Cortex.

[86] Peel H.J., Chouinard P.A. (2022). FMRI Form Adaptation and Size Repetition Enhancement in Different Subdivisions of the Lateral Occipital Complex. Cortex.

[87] Henson R., Shallice T., Dolan R. (2000). Neuroimaging Evidence for Dissociable Forms of Repetition Priming. Science.

[88] Ishai A., Bikle P.C., Ungerleider L.G. (2006). Temporal Dynamics of Face Repetition Suppression. Brain Res. Bull..

[89] Todorovic A., de Lange F.P. (2012). Repetition Suppression and Expectation Suppression Are Dissociable in Time in Early Auditory Evoked Fields. J. Neurosci..

[90] van Turennout M. (2003). Modulation of Neural Activity during Object Naming: Effects of Time and Practice. Cereb. Cortex.

[91] van Turennout M., Ellmore T., Martin A. (2000). Long-Lasting Cortical Plasticity in the Object Naming System. Nat. Neurosci..

[92] Lange K. (2011). The Reduced N1 to Self-Generated Tones: An Effect of Temporal Predictability?: N1 Reduction and Temporal Predictability. Psychophysiology.

[93] Friston K. (2005). A Theory of Cortical Responses. Phil. Trans. R. Soc..

[94] Grotheer M., Kovács G. (2016). Can Predictive Coding Explain Repetition Suppression?. Cortex.

[95] Wacongne C., Labyt E., van Wassenhove V., Bekinschtein T., Naccache L., Dehaene S. (2011). Evidence for a Hierarchy of Predictions and Prediction Errors in Human Cortex. Proc. Natl. Acad. Sci. USA.

[96] Tassinari H., Hudson T.E., Landy M.S. (2006). Combining Priors and Noisy Visual Cues in a Rapid Pointing Task. J. Neurosci..

[97] Tong J., Ngo V., Goldreich D. (2016). Tactile Length Contraction as Bayesian Inference. J. Neurophysiol..

[98] Clark A. (2013). Whatever next? Predictive Brains, Situated Agents, and the Future of Cognitive Science. Behav. Brain Sci..

[99] Friston K. (2010). The Free-Energy Principle: A Unified Brain Theory?. Nat. Rev. Neurosci..

[100] Feldman H., Friston K.J. (2010). Attention, Uncertainty, and Free-Energy. Front. Hum. Neurosci..

[101] Bendixen A., SanMiguel I., Schröger E. (2012). Early Electrophysiological Indicators for Predictive Processing in Audition: A Review. Int. J. Psychophysiol..

[102] Winkler I. (2007). Interpreting the Mismatch Negativity. J. Psychophysiol..

[103] Winkler I., Czigler I. (1998). Mismatch Negativity: Deviance Detection or the Maintenance of the “Standard”. Neuroreport.

[104] Mindell J.A., Williamson A.A. (2018). Benefits of a Bedtime Routine in Young Children: Sleep, Development, and Beyond. Sleep Med. Rev..

[105] Rodger S., Umaibalan V. (2011). The Routines and Rituals of Families of Typically Developing Children Compared with Families of Children with Autism Spectrum Disorder: An Exploratory Study. Br. J. Occup. Ther..

[106] Nakano T., Watanabe H., Homae F., Taga G. (2009). Prefrontal Cortical Involvement in Young Infants’ Analysis of Novelty. Cereb. Cortex.

[107] Turk-Browne N.B., Scholl B.J., Chun M.M. (2008). Babies and Brains: Habituation in Infant Cognition and Functional Neuroimaging. Front. Hum. Neurosci..

[108] Kulig J.W., Tighe T.J. (1981). Habituation in Children within a Behavior Suppression Paradigm. J. Exp. Child Psychol..

[109] Nguyen L.Y., Spehar B. (2021). Visual Adaptation to Natural Scene Statistics and Visual Preference. Vis. Res..

[110] Fantz R.L. (1964). visual experience in infants: Decreased attention to familiar patterns relative to novel ones. Science.

[111] Nordt M., Hoehl S., Weigelt S. (2016). The Use of Repetition Suppression Paradigms in Developmental Cognitive Neuroscience. Cortex.

[112] Fagan J.F. (1972). Infants’ Recognition Memory for Faces. J. Exp. Child Psychol..

[113] Hunter M.A., Ames E.W. (1988). A Multifactor Model of Infant Preferences for Novel and Familiar Stimuli. Advances in Infancy Research.

[114] Dehaene-Lambertz G., Dehaene S., Anton J.-L., Campagne A., Ciuciu P., Dehaene G.P., Denghien I., Jobert A., LeBihan D., Sigman M. (2006). Functional Segregation of Cortical Language Areas by Sentence Repetition. Hum. Brain Mapp..

[115] Bouchon C., Nazzi T., Gervain J. (2015). Hemispheric Asymmetries in Repetition Enhancement and Suppression Effects in the Newborn Brain. PLoS ONE.

[116] Martineau J., Roux S., Garreau B., Adrien J.L., Lelord G. (1992). Unimodal and Crossmodal Reactivity in Autism: Presence of Auditory Evoked Responses and Effect of the Repetition of Auditory Stimuli. Biol. Psychiatry.

[117] von Koss Torkildsen J., Hansen H.F., Svangstu J.M., Smith L., Simonsen H.G., Moen I., Lindgren M. (2009). Brain Dynamics of Word Familiarization in 20-Month-Olds: Effects of Productive Vocabulary Size. Brain Lang..

[118] Garrido M.I., Kilner J.M., Kiebel S.J., Stephan K.E., Baldeweg T., Friston K.J. (2009). Repetition Suppression and Plasticity in the Human Brain. Neuroimage.

[119] Gorina-Careta N., Zarnowiec K., Costa-Faidella J., Escera C. (2016). Timing Predictability Enhances Regularity Encoding in the Human Subcortical Auditory Pathway. Sci. Rep..

[120] Fryer S.L., Roach B.J., Hamilton H.K., Bachman P., Belger A., Carrión R.E., Duncan E., Johannesen J., Light G.A., Niznikiewicz M. (2020). Deficits in Auditory Predictive Coding in Individuals with the Psychosis Risk Syndrome: Prediction of Conversion to Psychosis. J. Abnorm. Psychol..

[121] Jamal W., Cardinaux A., Haskins A.J., Kjelgaard M., Sinha P. (2020). Reduced Sensory Habituation in Autism and Its Correlation with Behavioral Measures. J. Autism Dev. Disord..

[122] Feuerriegel D., Yook J., Quek G.L., Hogendoorn H., Bode S. (2021). Visual Mismatch Responses Index Surprise Signalling but Not Expectation Suppression. Cortex.

[123] Alho K., Sainio K., Sajaniemi N., Reinikainen K., Näätänen R. (1990). Event-Related Brain Potential of Human Newborns to Pitch Change of an Acoustic Stimulus. Electroencephalogr. Clin. Neurophysiol..

[124] Dehaene-Lambertz G., Dehaene S. (1994). Speed and Cerebral Correlates of Syllable Discrimination in Infants. Nature.

[125] Cheour M., Alho K., Ceponiené R., Reinikainen K., Sainio K., Pohjavuori M., Aaltonen O., Näätänen R. (1998). Maturation of Mismatch Negativity in Infants. Int. J. Psychophysiol..

[126] Rapaport H., Seymour R.A., Benikos N., He W., Pellicano E., Brock J., Sowman P.F. (2023). Investigating Predictive Coding in Younger and Older Children Using MEG and a Multi-Feature Auditory Oddball Paradigm. Cereb. Cortex.

[127] Gomot M., Giard M.-H., Roux S., Barthélémy C., Bruneau N. (2000). Maturation of Frontal and Temporal Components of Mismatch Negativity (MMN) in Children. NeuroReport.

[128] Cheour M., Leppänen P.H., Kraus N. (2000). Mismatch Negativity (MMN) as a Tool for Investigating Auditory Discrimination and Sensory Memory in Infants and Children. Clin. Neurophysiol..

[129] Charpentier J., Kovarski K., Houy-Durand E., Malvy J., Saby A., Bonnet-Brilhault F., Latinus M., Gomot M. (2018). Emotional Prosodic Change Detection in Autism Spectrum Disorder: An Electrophysiological Investigation in Children and Adults. J. Neurodev. Disord..

[130] Charpentier J., Kovarski K., Roux S., Houy-Durand E., Saby A., Bonnet-Brilhault F., Latinus M., Gomot M. (2018). Brain Mechanisms Involved in Angry Prosody Change Detection in School-Age Children and Adults, Revealed by Electrophysiology. Cogn. Affect. Behav. Neurosci..

[131] Chen T.-C., Hsieh M.H., Lin Y.-T., Chan P.-Y.S., Cheng C.-H. (2020). Mismatch Negativity to Different Deviant Changes in Autism Spectrum Disorders: A Meta-Analysis. Clin. Neurophysiol..

[132] Cléry H., Bonnet-Brilhault F., Lenoir P., Barthelemy C., Bruneau N., Gomot M. (2013). Atypical Visual Change Processing in Children with Autism: An Electrophysiological Study. Psychophysiology.

[133] Gomot M., Giard M.-H., Adrien J.-L., Barthelemy C., Bruneau N. (2002). Hypersensitivity to Acoustic Change in Children with Autism: Electrophysiological Evidence of Left Frontal Cortex Dysfunctioning. Psychophysiology.

[134] Gomot M., Blanc R., Clery H., Roux S., Barthelemy C., Bruneau N. (2011). Candidate Electrophysiological Endophenotypes of Hyper-Reactivity to Change in Autism. J. Autism Dev. Disord..

[135] Kovarski K., Charpentier J., Roux S., Batty M., Houy-Durand E., Gomot M. (2021). Emotional Visual Mismatch Negativity: A Joint Investigation of Social and Non-Social Dimensions in Adults with Autism. Transl. Psychiatry.

[136] Franken I.H.A., Nijs I., Van Strien J.W. (2005). Impulsivity Affects Mismatch Negativity (MMN) Measures of Preattentive Auditory Processing. Biol. Psychol..

[137] Rydkjær J., Jepsen J.R.M., Pagsberg A.K., Fagerlund B., Glenthøj B.Y., Oranje B. (2017). Mismatch Negativity and P3a Amplitude in Young Adolescents with First-Episode Psychosis: A Comparison with ADHD. Psychol. Med..

[138] Yang M.-T., Hsu C.-H., Yeh P.-W., Lee W.-T., Liang J.-S., Fu W.-M., Lee C.-Y. (2015). Attention Deficits Revealed by Passive Auditory Change Detection for Pure Tones and Lexical Tones in ADHD Children. Front. Hum. Neurosci..

[139] Chen C., Sung J.-Y., Cheng Y. (2016). Neural Dynamics of Emotional Salience Processing in Response to Voices during the Stages of Sleep. Front. Behav. Neurosci..

[140] Baldeweg T., Hirsch S.R. (2015). Mismatch Negativity Indexes Illness-Specific Impairments of Cortical Plasticity in Schizophrenia: A Comparison with Bipolar Disorder and Alzheimer’s Disease. Int. J. Psychophysiol..

[141] Erickson M.A., Ruffle A., Gold J.M. (2016). A Meta-Analysis of Mismatch Negativity in Schizophrenia: From Clinical Risk to Disease Specificity and Progression. Biol. Psychiatry.

[142] Kirihara K., Tada M., Koshiyama D., Fujioka M., Usui K., Araki T., Kasai K. (2020). A Predictive Coding Perspective on Mismatch Negativity Impairment in Schizophrenia. Front. Psychiatry.

[143] Näätänen R., Kähkönen S. (2009). Central Auditory Dysfunction in Schizophrenia as Revealed by the Mismatch Negativity (MMN) and Its Magnetic Equivalent MMNm: A Review. Int. J. Neuropsychopharm..

[144] American Psychiatric Association (2013). Diagnostic and Statistical Manual of Mental Disorders.

[145] Baranek G.T., Barnett C.R., Adams E.M., Wolcott N.A., Watson L.R., Crais E.R. (2005). Object Play in Infants with Autism: Methodological Issues in Retrospective Video Analysis. Am. J. Occup. Ther..

[146] Liss M., Saulnier C., Fein D., Kinsbourne M. (2006). Sensory and Attention Abnormalities in Autistic Spectrum Disorders. Autism.

[147] Rogers S.J., Hepburn S., Wehner E. (2003). Parent Reports of Sensory Symptoms in Toddlers with Autism and Those with Other Developmental Disorders. J. Autism Dev. Disord..

[148] Gomot M., Wicker B. (2012). A Challenging, Unpredictable World for People with Autism Spectrum Disorder. Int. J. Psychophysiol. Off. J. Int. Organ. Psychophysiol..

[149] Brock J. (2012). Alternative Bayesian Accounts of Autistic Perception: Comment on Pellicano and Burr. Trends Cogn. Sci..

[150] Karaminis T., Cicchini G.M., Neil L., Cappagli G., Aagten-Murphy D., Burr D., Pellicano E. (2016). Central Tendency Effects in Time Interval Reproduction in Autism. Sci. Rep..

[151] Crespo C., Santos S., Canavarro M.C., Kielpikowski M., Pryor J., Féres-Carneiro T. (2013). Family Routines and Rituals in the Context of Chronic Conditions: A Review. Int. J. Psychol..

[152] Rogers S.J., Ozonoff S. (2005). Annotation: What Do We Know about Sensory Dysfunction in Autism? A Critical Review of the Empirical Evidence. J. Child Psychol. Psychiatry.

[153] Cary E., Pacheco D., Kaplan-Kahn E., McKernan E., Matsuba E., Prieve B., Russo N. (2023). Brain Signatures of Early and Late Neural Measures of Auditory Habituation and Discrimination in Autism and Their Relationship to Autistic Traits and Sensory Overresponsivity. J. Autism Dev. Disord..

[154] Gonzalez-Gadea M.L., Chennu S., Bekinschtein T.A., Rattazzi A., Beraudi A., Tripicchio P., Moyano B., Soffita Y., Steinberg L., Adolfi F. (2015). Predictive Coding in Autism Spectrum Disorder and Attention Deficit Hyperactivity Disorder. J. Neurophysiol..

[155] Guiraud J.A., Kushnerenko E., Tomalski P., Davies K., Ribeiro H., Johnson M.H. (2011). Differential Habituation to Repeated Sounds in Infants at High Risk for Autism. NeuroReport.

[156] Font-Alaminos M., Cornella M., Costa-Faidella J., Hervás A., Leung S., Rueda I., Escera C. (2020). Increased Subcortical Neural Responses to Repeating Auditory Stimulation in Children with Autism Spectrum Disorder. Biol. Psychol..

[157] Latinus M., Cléry H., Andersson F., Bonnet-Brilhault F., Fonlupt P., Gomot M. (2019). Inflexibility in Autism Spectrum Disorder: Need for Certainty and Atypical Emotion Processing Share the Blame. Brain Cogn..

[158] Rubenstein J.L.R., Merzenich M.M. (2003). Model of Autism: Increased Ratio of Excitation/Inhibition in Key Neural Systems. Genes Brain Behav..

[159] Briend F., Barantin L., Cléry H., Cottier J.-P., Bonnet-Brilhault F., Houy-Durand E., Gomot M. (2023). Glutamate Levels of the Right and Left Anterior Cingulate Cortex in Autistics Adults. Prog. Neuro-Psychopharmacol. Biol. Psychiatry.

[160] Galineau L., Arlicot N., Dupont A.-C., Briend F., Houy-Durand E., Tauber C., Gomot M., Gissot V., Barantin L., Lefevre A. (2023). Glutamatergic Synapse in Autism: A Complex Story for a Complex Disorder. Mol. Psychiatry.

[161] Sapey-Triomphe L.-A., Temmerman J., Puts N.A.J., Wagemans J. (2021). Prediction Learning in Adults with Autism and Its Molecular Correlates. Mol. Autism.

[162] Kolesnik A., Begum Ali J., Gliga T., Guiraud J., Charman T., Johnson M.H., Jones E.J.H. (2019). Increased Cortical Reactivity to Repeated Tones at 8 Months in Infants with Later ASD. Transl. Psychiatry.

[163] Friston K.J., Lawson R., Frith C.D. (2013). On Hyperpriors and Hypopriors: Comment on Pellicano and Burr. Trends Cogn. Sci..

[164] Lawson R.P., Rees G., Friston K.J. (2014). An Aberrant Precision Account of Autism. Front. Hum. Neurosci..

[165] Van de Cruys S., Evers K., Van der Hallen R., Van Eylen L., Boets B., de-Wit L., Wagemans J. (2014). Precise Minds in Uncertain Worlds: Predictive Coding in Autism. Psychol. Rev..

[166] Sinha P., Kjelgaard M.M., Gandhi T.K., Tsourides K., Cardinaux A.L., Pantazis D., Diamond S.P., Held R.M. (2014). Autism as a Disorder of Prediction. Proc. Natl. Acad. Sci. USA.

[167] Cannon J., O’Brien A.M., Bungert L., Sinha P. (2021). Prediction in Autism Spectrum Disorder: A Systematic Review of Empirical Evidence. Autism Res..

[168] Randeniya R., Mattingley J.B., Garrido M.I. (2022). Increased Context Adjustment Is Associated with Auditory Sensitivities but Not with Autistic Traits. Autism Res..

[169] Kleinhans N.M., Johnson L.C., Richards T., Mahurin R., Greenson J., Dawson G., Aylward E. (2009). Reduced Neural Habituation in the Amygdala and Social Impairments in Autism Spectrum Disorders. Am. J. Psychiatry.

[170] D’Mello A.M., Frosch I.R., Meisler S.L., Grotzinger H., Perrachione T.K., Gabrieli J.D.E. (2023). Diminished Repetition Suppression Reveals Selective and Systems-Level Face Processing Differences in ASD. J. Neurosci..

[171] Millin R., Kolodny T., Flevaris A.V., Kale A.M., Schallmo M.-P., Gerdts J., Bernier R.A., Murray S. (2018). Reduced Auditory Cortical Adaptation in Autism Spectrum Disorder. eLife.

[172] Lawson R.P., Aylward J., Roiser J.P., Rees G. (2018). Adaptation of Social and Non-Social Cues to Direction in Adults with Autism Spectrum Disorder and Neurotypical Adults with Autistic Traits. Dev. Cogn. Neurosci..

[173] Hemsley D.R. (2005). The Development of a Cognitive Model of Schizophrenia: Placing It in Context. Neurosci. Biobehav. Rev..

[174] Sterzer P., Adams R.A., Fletcher P., Frith C., Lawrie S.M., Muckli L., Petrovic P., Uhlhaas P., Voss M., Corlett P.R. (2018). The Predictive Coding Account of Psychosis. Biol. Psychiatry.

[175] Thakkar K.N., Silverstein S.M., Brascamp J.W. (2019). A Review of Visual Aftereffects in Schizophrenia. Neurosci. Biobehav. Rev..

[176] Bolino F., Di Michele V., Di Cicco L., Manna V., Daneluzzo E., Casacchia M. (1994). Sensorimotor Gating and Habituation Evoked by Electro-Cutaneous Stimulation in Schizophrenia. Biol. Psychiatry.

[177] Freedman R. (1996). Inhibitory Gating of an Evoked Response to Repeated Auditory Stimuli in Schizophrenic and Normal Subjects: Human Recordings, Computer Simulation, and an Animal Model. Arch. Gen. Psychiatry.

[178] Avery S.N., McHugo M., Armstrong K., Blackford J.U., Vandekar S., Woodward N.D., Heckers S. (2020). Habituation during Encoding: A New Approach to the Evaluation of Memory Deficits in Schizophrenia. Schizophr. Res..

[179] Holt D.J., Weiss A.P., Rauch S.L., Wright C.I., Zalesak M., Goff D.C., Ditman T., Welsh R.C., Heckers S. (2005). Sustained Activation of the Hippocampus in Response to Fearful Faces in Schizophrenia. Biol. Psychiatry.

[180] Bodatsch M., Brockhaus-Dumke A., Klosterkötter J., Ruhrmann S. (2015). Forecasting Psychosis by Event-Related Potentials—Systematic Review and Specific Meta-Analysis. Biol. Psychiatry.

[181] Coffman B.A., Haigh S.M., Murphy T.K., Salisbury D.F. (2017). Impairment in Mismatch Negativity but Not Repetition Suppression in Schizophrenia. Brain Topogr..

[182] Koshiyama D., Kirihara K., Tada M., Nagai T., Fujioka M., Usui K., Araki T., Kasai K. (2020). Reduced Auditory Mismatch Negativity Reflects Impaired Deviance Detection in Schizophrenia. Schizophr. Bull..

[183] Rentzsch J., Shen C., Jockers-Scherübl M.C., Gallinat J., Neuhaus A.H. (2015). Auditory Mismatch Negativity and Repetition Suppression Deficits in Schizophrenia Explained by Irregular Computation of Prediction Error. PLoS ONE.

[184] Mazer P., Macedo I., Paiva T.O., Ferreira-Santos F., Pasion R., Barbosa F., Almeida P., Silveira C., Cunha-Reis C., Marques-Teixeira J. (2021). Abnormal Habituation of the Auditory Event-Related Potential P2 Component in Patients With Schizophrenia. Front. Psychiatry.

[185] McCleery A., Wynn J.K., Green M.F. (2018). Hallucinations, Neuroplasticity, and Prediction Errors in Schizophrenia. Scand. J. Psychol..

[186] Kovács G., Grotheer M., Münke L., Kéri S., Nenadić I. (2019). Significant Repetition Probability Effects in Schizophrenia. Psychiatry Res. Neuroimaging.

[187] Williams L.E., Blackford J.U., Luksik A., Gauthier I., Heckers S. (2013). Reduced Habituation in Patients with Schizophrenia. Schizophr. Res..

[188] Lee J., Reavis E.A., Engel S.A., Altshuler L.L., Cohen M.S., Glahn D.C., Nuechterlein K.H., Wynn J.K., Green M.F. (2019). FMRI Evidence of Aberrant Neural Adaptation for Objects in Schizophrenia and Bipolar Disorder. Hum. Brain Mapp..

[189] Jansiewicz E.M., Newschaffer C.J., Denckla M.B., Mostofsky S.H. (2004). Impaired Habituation in Children with Attention Deficit Hyperactivity Disorder. Cogn. Behav. Neurol..

[190] Massa J., O’Desky I.H. (2011). Impaired Visual Habituation in Adults With ADHD. J. Atten. Disord..

[191] Conzelmann A., Pauli P., Mucha R.F., Jacob C.P., Gerdes A.B.M., Romanos J., Bähne C.G., Heine M., Boreatti-Hümmer A., Alpers G.W. (2010). Early Attentional Deficits in an Attention-to-Prepulse Paradigm in ADHD Adults. J. Abnorm. Psychol..

[192] Herpertz S.C., Mueller B., Wenning B., Qunaibi M., Lichterfeld C., Herpertz-Dahlmann B. (2003). Autonomic Responses in Boys with Externalizing Disorders. J. Neural Transm..

[193] Lloyd D.R., Medina D.J., Hawk L.W., Fosco W.D., Richards J.B. (2014). Habituation of Reinforcer Effectiveness. Front. Integr. Neurosci..

[194] Friston K. (2009). The Free-Energy Principle: A Rough Guide to the Brain?. Trends Cogn. Sci..

[195] Cheng C.-H., Chan P.-Y.S., Hsieh Y.-W., Chen K.-F. (2016). A Meta-Analysis of Mismatch Negativity in Children with Attention Deficit-Hyperactivity Disorders. Neurosci. Lett..

[196] Kanner L. (1943). Autistic Disturbances of Affective Contact. Nerv. Child.

[197] Andronikof A., Fontan P. (2016). Grounia Efimovna Soukhareva: La Première Description Du Syndrome Dit d’Asperger. Neuropsychiatr. L’enfance L’adolescence.

